# Reassortant Clade 2.3.4.4 Avian Influenza A(H5N6) Virus in a Wild Mandarin Duck, South Korea, 2016

**DOI:** 10.3201/eid2305.161905

**Published:** 2017-05

**Authors:** Jung-Hoon Kwon, Dong-Hun Lee, David E. Swayne, Jin-Yong Noh, Seong-Su Yuk, Tseren-Ochir Erdene-Ochir, Woo-Tack Hong, Jei-Hyun Jeong, Sol Jeong, Gyeong-Bin Gwon, Seok Lee, Chang-Seon Song

**Affiliations:** Konkuk University, Seoul, South Korea (J.-H. Kwon, J.-Y. Noh, S.-S. Yuk, T.-O. Erdene-Ochir, W.-T. Hong, J.-H. Jeong, S. Jeong, G.-B. Gwon, C.-S. Song);; US Department of Agriculture, Athens, Georgia, USA (D.-H. Lee, D.E. Swayne);; Korea Institute of Science and Technology, Seoul (S. Lee)

**Keywords:** influenza, influenza virus, highly pathogenic avian influenza virus, HPAIV, H5N6 subtype, clade 2.3.4.4, viruses, reassortant, wild birds, mandarin duck, Aix galericulata, respiratory infections, zoonoses, South Korea

## Abstract

A reassortant clade 2.3.4.4 avian influenza A(H5N6) virus was isolated from a fecal sample of a Mandarin duck (*Aix galericulata*) in South Korea during October 2016. This virus was genetically similar to H5N6 subtype virus isolates from China, Vietnam, Laos, and Hong Kong, including human isolates.

Highly pathogenic avian influenza viruses (HPAIVs) have caused major economic losses in poultry industries and represent a serious threat to public health. The H5N1 subtype of these viruses was first detected in 1996 from a domestic goose in Guangdong, China (Gs/GD), and its H5 hemagglutinin (HA) gene has subsequently evolved into 10 genetically distinct virus clades (0–9) and multiple subclades (*1*). Since 2008, novel reassortant HPAIVs bearing the HA gene of the Gs/GD lineage H5 clade 2.3.4 and neuraminidase (NA) gene subtypes N1, N2, N5, N6, N8, and N9 have been identified in China (*2*).

Although clade 2.3.4 of influenza A(H5N8) virus caused influenza outbreaks in eastern Asia and was subsequently disseminated into Europe and North America by wild aquatic birds in late 2014 (*3*,*4*), clade 2.3.4.4 of this virus has caused continuous outbreaks in China since 2013 (*5*). This virus disseminated into Laos and Vietnam in 2014 and Hong Kong in 2015 (*6*,*7*). Since the first influenza case in Sichuan Province, China, 15 human cases of influenza caused by this subtype have been reported in China during April 2014–May 2016 (*8*).

We report detection of an H5N6 subtype HPAIV in a fecal sample obtained from a wild bird sampled in South Korea during the fall 2016. We sequenced and genetically analyzed the complete genome of this virus isolate.

## The Study

On October 28, 2016, we isolated an H5N6 subtype HPAIV from 1 of 391 fecal samples collected from wild birds in Gokgyo-cheon, South Korea (36°45′12.3″N, 127°07′12.7″E). Gokgyo-cheon is a wild bird habitat for wintering of migratory waterfowl, including mallard (*Anas platyrhynchos*), spot-billed duck (*Anas poecilorhyncha*), Mandarin duck (*Aix galericulata*), and common teal (*Anas crecca*). The species of the positive fecal sample was identified as Mandarin duck on the basis of DNA barcoding technique as described (*9*). There were no detectable clinically ill or dead wild birds at the sampling site.

Full-length genome sequencing and phylogenetic analysis were conducted to trace the origin of A/Mandarin_duck/Korea/K16-187-3/2016(H5N6) virus, hereafter referred to as MD/KR/2016. Methods used are detailed in Technical Appendix 1. We entered genome sequences in the GISAID (Global Initiative on Sharing All Influenza Data) EpiFlu database (https://www.gisaid.org) under accession nos. EPI861480–EPI861488. Strains used in analysis are shown in Technical Appendix 2.

The isolate was identified as an HPAIV on the basis of multiple basic amino acids at the HA proteolytic cleavage site (PLRERRRKR/G). GISAID BLAST (https://blast.ncbi.nlm.nih.gov/Blast.cgi) searches indicated that H5 and N6 genes had high nucleotide identity in HA (99.17%) and NA (99.24%) with A/great_egret/Hong_Kong/00032/2016 (H5N6) (Table 1). Internal gene segments, except the polymerase basic 1 (PB1) gene, had high nucleotide identity with other H5N6 subtypes isolated in Guangdong and Jiangxi, China (PB2, 99.09%; polymerase acidic, 98.96%; nucleoprotein, 99.16%; matrix, 98.98%; and nonstructural protein [NS], 98.31%). However, the PB1 gene had high nucleotide identity (97.01%) with H4 low pathogenicity avian influenza viruses (LPAIVs).

**Table 1:** Nucleotide identities between reassortant clade 2.3.4.4 avian influenza A(H5N6) virus isolated from a wild Mandarin duck, South Korea, 2016, and nearest virus homologs in the GISAID database*

Gene	Virus	GISAID accession no.	% Identity
PB2	A/feline/Guangdong/2/2015(H5N6)	EPI760095	99.09
PB1	A/duck/Guangdong/S4040/2011(H4N2)	EPI692414	97.01
PA	A/Syrrhaptes paradoxus/Guangdong/ZH283/2015(H5N6)	EPI839169	98.96
HA	A/great_egret/Hong_Kong/00032/2016(H5N6)	EPI687156	99.17
NP	A/Syrrhaptes paradoxus/Guangdong/ZH283/2015(H5N6)	EPI839171	99.16
NA	A/great_egret/Hong_Kong/00032/2016(H5N6)	EPI687157	99.24
M	A/feline/Guangdong/2/2015(H5N6)	EPI760101	98.98
NS	A/duck/Jiangxi/NCDZT1123/2014(H5N6)	EPI590810	98.31

*GISAID, Global Initiative on Sharing All Influenza Data (http://www.gisaid.org); HA, hemagglutinin; MP, matrix; NA, neuraminidase; NP, nucleoprotein; NS, nonstructural protein; PA, polymerase acidic; PB1, polymerase basic 1; PB2, polymerase basic 2.

In previous phylogenetic analyses, the HA gene of clade 2.3.4.4 viruses was divided into 4 distinct subgroups (Technical Appendix 1 Figure 1) (*10*). Group intercontinental A (icA) contains H5N8 subtype virus and its reassortant viruses identified in China, South Korea, Japan, Taiwan, Canada, the United States, and countries in Europe during 2013–2016. Group B contains H5N8 subtype viruses identified in China and South Korea during 2013–2014, and in Russia in late 2016. Group C contains H5N1 and H5N6 subtype viruses identified in China, Vietnam, Laos, and Hong Kong, including isolates from humans in Guangdong, Yunnan, and Hunnan Provinces, China. Group D contains H5N6 subtype viruses identified in China and Vietnam, including an isolate from a human in Sichuan Province, China. The HA gene of MD/KR/2016 virus belonged to group C and clustered with H5N6 subtype viruses isolated from humans, cats, and the environment in Guangdong during 2014–2015 and a migratory aquatic bird in Hong Kong during January 2016 (A/great_egret/Hong_Kong/00032/2016 [H5N6]) (Technical Appendix 1 Figure 1).

A previous study reported that A/environment/Guangdong/GZ693/2015 (H5N6), hereafter referred to as GZ693/2015(H5N6), is a 7:1 gene reassortant virus between H5N6 HPAIV and LPAIVs found in southern China (*7*). MD/KR/2016 clustered with GZ693/2015(H5N6) virus for all 8 genes (Technical Appendix 1 Figure 2). In particular, the HA, NA, PB2, polymerase acidic, nucleoprotein, matrix, and NS protein genes clustered with GZ693/2015(H5N6) and other clade 2.3.4.4 group C H5N6 viruses. The PB1 gene clustered with GZ693/2015(H5N6) (nucleotide identity 92.79%) and LPAIVs, such as H3N2 and H4N2 subtype viruses, from southern China. Phylogenetic analysis and BLAST search collectively suggest that MD/KR/2016 virus had an identical genotype to GZ693/2015(H5N6).

Most of clade 2.3.4.4 group C viruses have leucine or serine at position 129 (H5 numbering) in HA protein. However, MD/KR/2016 had a single amino acid deletion at position 129 (Table 2), as did A/great_egret/Hong_Kong/00032/2016 (H5N6). This deletion at position 129 and phylogenetic network analysis suggested that MD/KR/2016 is closely related to H5N6 subtypes isolated from wild birds in Hong Kong in 2016 (Technical Appendix 1 Figure 3). MD/KR/2016 contained the mutation associated with increased virulence in mammals and mammalian transmissibility (S123P and T156A mutations in the HA gene; P42S and D92E mutation, and elongated C-terminus with PDZ binding motif in NS gene). However, this isolate lacked the Q226L and G228S mutations in HA, which have been associated with increased binding to human-type receptor (α-2,6–linked sialic acid) and lacked Q591K, E627K and D701N mutations in PB2, which have been associated with enhanced pathogenicity and adaptation to mammalian hosts (*11*). All of the 9 H5N6 subtype human isolates of group C lacked the Q226L and G228S mutations in HA, but 5 viruses contained the E627K mutation in PB2 (Table 2), suggesting that some purported mammalian adaptation amino acid substitutions were not necessary for sporadic virus infection of H5N6 HPAIV in humans.

**Table 2:** Amino acid analysis of avian influenza A(H5N6) virus from a wild mandarin duck, South Korea, 2016, and reference strains of clade 2.3.4.4 H5N6 subtype virus*

Group, strain																		NA del,§ 59–69	NS¶		
	HA (H5 numbering)†														PB2‡				42	80–84 del	PDZ
	123	126		129		133		156		222		224			591	627	701				
South Korea H5N6 subtype and closely related avian isolates																					
A/Mandarin_duck/Korea/K16-187-3/ 2016	P		E		Del		S		A		Q		G		Q	E	D	Yes	S	Yes	ESEV
A/great_egret/Hong_Kong/00032/2016	P		E		Del		A		A		Q		G		Q	?	?	Yes	?	?	No
A/environment/Guangdong/GZ693/ 2015	P		E		L		A		A		Q		G		Q	E	D	No	S	No	ESEV
C, human isolates																					
A/Shenzhen/1/2016	P		Del		S		A		A		Q		G		Q	K	D	Yes	S	No	No
A/_Guangdong_/ZQ874/2015H5N6	P		E		L		A		A		Q		G		Q	E	D	Yes	S	Del	ESEV
A/_Guangdong_/SZ872/2015H5N6	P		Del		S		A		A		Q		G		Q	E	D	Yes	S	No	No
A/Shenzhen/1/2015	P		Del		S		A		A		Q		G		Q	E	D	Yes	S	No	No
A/Yunnan/14563/2015	P		Del		S		A		A		Q		G		Q	K	D	Yes	S	No	No
A/Yunnan/14564/2015	P		Del		S		A		A		Q		G		Q	K	D	Yes	S	No	No
A/Yunnan/0127/2015	P		Del		S		A		A		Q		G		Q	K	D	Yes	S	Yes	No
A/Guangzhou/39715/2014	P		E		L		A		T		Q		G		Q	K	D	Yes	S	Yes	ESEV
A/Changsha/1/2014	P		Del		S		A		A		Q		G		Q	E	D	Yes	S	Yes	ESEV
D, human isolate																					
A/Sichuan/26221/2014	T		E		L		A		A		Q		G		Q	E	N	No	S	Yes	ESEV
C, mammalian isolates																					
A/swine/Guangdong/1/2014	P		E		L		A		A		Q		G		Q	E	D	Yes	S	Yes	EPEV
A/swine/Guangdong/2/2014	P		E		L		A		A		Q		G		Q	E	D	Yes	S	Yes	EPEV
A/feline/Guangdong/1/2015	P		E		L		A		A		Q		G		Q	E	D	Yes	S	Yes	ESEV
A/feline/Guangdong/2/2015	P		E		L		A		A		Q		G		Q	E	D	Yes	S	Yes	ESEV

*Del, deletion; HA, hemagglutinin; NA, neuraminidase; NS, nonstructural protein; PB2, polymerase basic 2; PDZ, PDZ binding motif. †S123P, S133A, T156A, Q222L, and G224S mutations in HA have been associated with increased binding to human-like receptor (α-2–6 sialic acid). ‡Q591K, E627K, and D701N mutations have been associated with improved replication of avian influenza virus in mammals. §NA stalk deletion has been associated with enhanced pathogenicity in mice. ¶42S, 80–84 deletion, and ESEV PDZ binding motif have been associated with increased virulence in mice.

## Conclusions

Wild aquatic birds have been suspected to play a key role in dissemination of HPAIVs to various regions, as seen with clade 2.2 H5N1 HPAIV in 2005, clade 2.3.2.1 H5N1 HPAIV in 2009, and clade 2.3.4.4 H5N8 HPAIV in 2014 (*4*,*12*). Some populations of Mandarin ducks are year-round residents in South Korea and Japan; others populations migrate between Russia and eastern Asia (*13*). In South Korea, HPAIV was detected from Mandarin duck samples in 2010 (H5N1) and 2014 (H5N8) (*14*,*15*) and again in 2016 during this study, suggesting that Mandarin ducks are a major host species for clade 2.3.4.4 H5 HPAIV and can disseminate the virus throughout South Korea and into other countries. Detection of the H5N6 HPAIV clade 2.3.4.4 in a migratory bird species in South Korea; reports of H5N6 outbreaks in poultry from China, Laos, and Vietnam; and diagnosis of lethal human cases of highly homologous H5N6 viruses in China raise a concern over the potential for broad geographic dissemination of zoonotic H5N6 HPAIV by wild birds outside eastern Asia.

Technical Appendix 1 Additional information on reassortant clade 2.3.4.4 avian influenza A(H5N6) virus in a wild mandarin duck, South Korea, 2016.

Technical Appendix 2 Influenza virus isolates used in the study of reassortant clade 2.3.4.4 avian influenza A(H5N6) virus in a wild mandarin duck, South Korea, 2016.
