# Supplementary material for: Reassortant Clade 2.3.4.4 Avian Influenza A(H5N6) Virus in a Wild Mandarin Duck, South Korea, 2016
Source: Emerg Infect Dis. 2017 May;23(5):822–6. doi: 10.3201/eid2305.161905 (PMC5403023; doi:10.3201/eid2305.161905)
Supplement: Technical Appendix 1 — Additional information on reassortant clade 2.3.4.4 avian influenza A(H5N6) virus in a wild mandarin duck, South Korea, 2016. [file 16-1905-Techapp-s1.pdf]

Article DOI: http://dx.doi.org/10.3201/eid2305.161905

Reassortant Clade 2.3.4.4 Avian Influenza A(H5N6) Virus in a Wild Mandarin Duck, South Korea, 2016

Technical Appendix 1

Methods

One of 391 fecal samples was positive for the influenza A virus by egg inoculation and matrix gene real-time reverse transcription PCR (cycle threshold 25.52) performed as described (*1*). The host of the positive fecal sample was identified as a mandarin duck (*Aix galericulata*) by using DNA barcoding techniques as described (*2*). Full-length genome sequencing of A/Mandarin_duck/Korea/K16–187–3/2016(H5N6) virus was performed by using conventional reverse transcription PCR and Sanger sequencing.

For phylogenetic analysis, nucleotide sequences identified in this study were deposited in GISAID (www.gisaid.org) and GenBank (www.ncbi.nlm.nih.gov/genomes/FLU). Complete coding regions were aligned by using MUSCLE (http://drive5.com/muscle/). Manual editing and tree reconstruction were performed by using MEGA7 (http://www.megasoftware.net). A maximum-likelihood tree was estimated by using MEGA7 and the Hasegawa–Kishino–Yano model of nucleotide substitution with gamma-distributed rate variation among sites with 4 rate categories.

Statistical analysis of the phylogenetic tree was performed by using bootstrap analysis performed for 1,000 replicates. A median-joining phylogenetic network of group C H5N6 viruses was constructed by using NETWORK version 5.0 with epsilon set to 0 (www.fluxus-engineering.com).

References

1. Spackman E, Senne DA, Bulaga LL, Myers TJ, Perdue ML, Garber LP, et al. Development of real-time RT-PCR for the detection of avian influenza virus. Avian Dis. 2003;47(Suppl):1079–82. PubMed http://dx.doi.org/10.1637/0005-2086-47.s3.1079

2. Lee DH, Lee HJ, Lee YJ, Kang HM, Jeong OM, Kim MC, et al. DNA barcoding techniques for avian influenza virus surveillance in migratory bird habitats. J Wildl Dis. 2010;46:649–54. PubMed http://dx.doi.org/10.7589/0090-3558-46.2.649

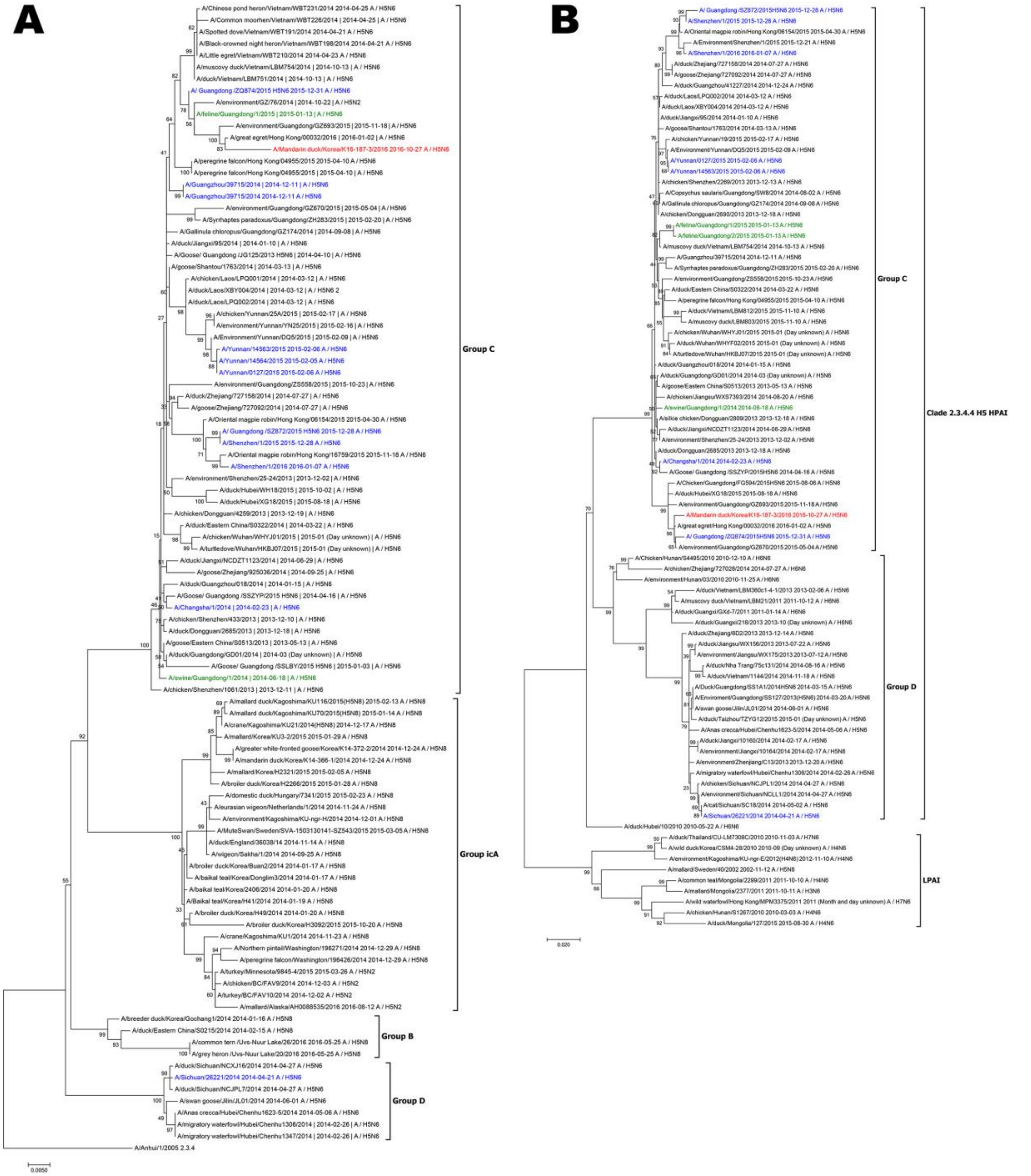

Technical Appendix Figure 1. Maximum-likelihood phylogenetic trees for A) hemagglutinin (HA) and B) neuraminidase (NA) genes of avian influenza viruses. Red indicates avian influenza A(H5N6) virus isolated in South Korea in this study, blue indicates human isolates, and green mammalian isolates. Percentages of replicate trees in which associated taxa clustered together in the bootstrap test (1,000 replicates) are shown next to branches. Scale bars indicate nucleotide substitutions per site. HPAI, highly pathogenic avian influenza; LPAI, low pathogenicity avian influenza.

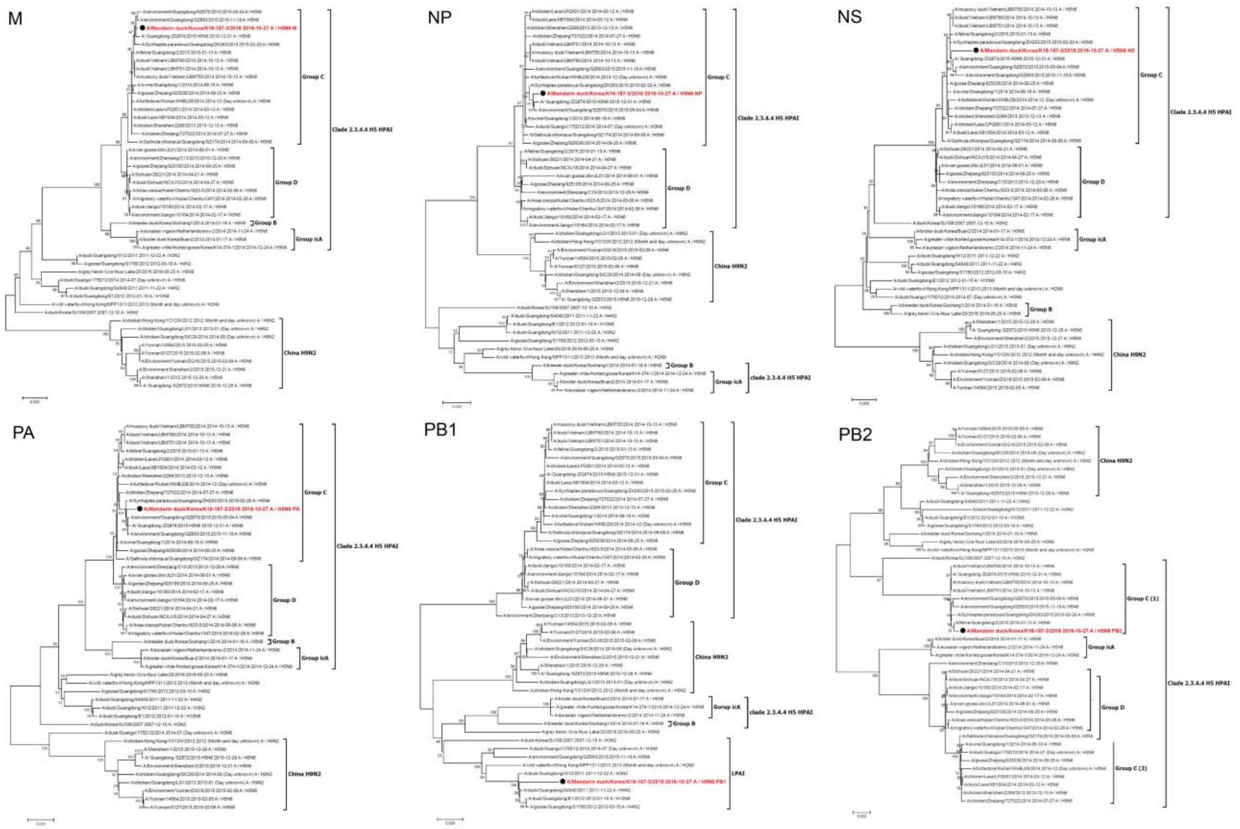

Technical Appendix Figure 2. Maximum-likelihood phylogenetic trees for polymerase basic 2 (PB2), PB1, acidic polymerase (PA), nucleoprotein (NP), matrix (M), and nonstructural protein (NS) genes of avian influenza viruses. Black circles and red indicate avian influenza A(H5N6) virus isolated in South Korea in this study. Percentages of replicate trees in which associated taxa clustered together in the bootstrap test (1,000 replicates) are shown next to branches. Scale bars indicate nucleotide substitutions per site. HPAI, highly pathogenic avian influenza; LPAI, low pathogenicity avian influenza.

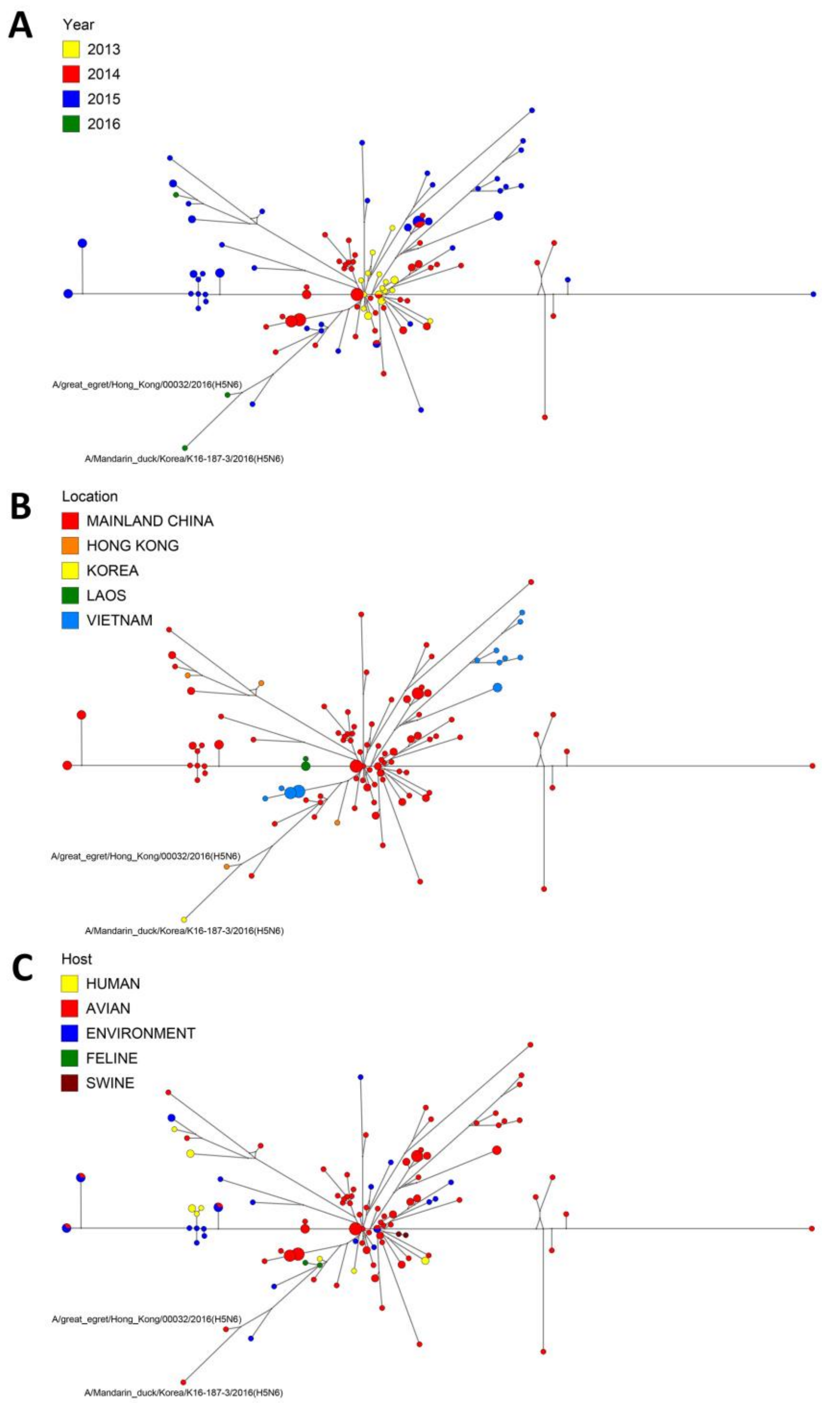

Technical Appendix Figure 3. Median-joining phylogenetic network of clade 2.3.4.4 avian influenza A(H5N6) viruses. The network was constructed from the hemagglutinin gene and includes all most parsimonious trees linking sequences. Each unique sequence is represented by a circle sized relative to its frequency in the dataset. Branch length is proportional to the number of mutations. Isolates are colored according to A) year of collection, B) location, and C) host.
